# Functional Flow Cytometric Assay for Reliable and Convenient Heparin-Induced Thrombocytopenia Diagnosis in Daily Practice

**DOI:** 10.3390/biomedicines9040332

**Published:** 2021-03-25

**Authors:** Brigitte Tardy-Poncet, Aurélie Montmartin, Michele Piot, Martine Alhenc-Gelas, Philippe Nguyen, Ismail Elalamy, Andreas Greinacher, Emmanuel De Maistre, Dominique Lasne, Marie-Hélène Horellou, Grégoire Le Gal, Thomas Lecompte, Bernard Tardy, on behalf of the GFHT-HIT Study Group

**Affiliations:** 1 Inserm U1059 Sainbiose, Université de Lyon, 42055 Saint-Etienne, France; aurelie.montmartin@univ-st-etienne.fr (A.M.); michele.piot@univ-st-etienne.fr (M.P.); bernard.tardy@yahoo.fr (B.T.); 2 Inserm CIC 1408, FCRIN-INNOVTE, Centre Hospitalier Universitaire de Saint-Etienne, 42000 Saint-Etienne, France; 3 Service d’Hématologie biologique, Hôpital Européen Georges Pompidou, AP-HP, 75000 Paris, France; martine.alhenc-gelas@aphp.fr; 4 Service d’Hématologie biologique, Pole de biologie, Hôpital Robert Debré, CHU de Reims, 51100 Reims, France; pnguyen@chu-reims.fr; 5 Centre d’Hématologie et de Thrombose, Hôpital Tenon, Inserm U938, Université Sorbonne, 75000 Paris, France; ismail.elalamy@aphp.fr; 6 Department of Obstetrics and Gynecology, I.M. Sechenov First Moscow State Medical University, 119435 Moscow, Russia; 7 Institut d’Hématologie et de Médecine transfusionnelle, Université de Greifswald, 17489 Greifswald, Germany; greinach@uni-greifswald.de; 8 Service d’Hématologie Biologique, Hôpital Le Bocage, CHRU de Dijon, 21000 Dijon, France; emmanuel.demaistre@chu-dijon.fr; 9 Service d’Hématologie Biologique, Hôpital Necker-Enfants Malades, AP-HP, 75000 Paris, France; dominique.lasne@aphp.fr; 10 Service d’Hématologie Biologique, Hôpital Universitaire Paris, 75000 Paris, France; marie.horellou@cch.aphp.fr; 11 Département d’Hématologie, Departement de Medicine, Institut de Recherché de l’hôpital d’Ottawa, Université d’Ottawa, Ottawa, ON K1P 1J1, Canada; glegal@ohri.ca; 12 Département de médecine, Hôpital Universitaire de Genève, Faculté de Médecine—Université de Genève, 57340 Genève, Switzerland; ThomasPierre.Lecompte@hcuge.ch

**Keywords:** heparin-induced thrombocytopenia, diagnosis, flow cytometry, serotonin release assay, expert opinion adjudication

## Abstract

Reliable laboratory diagnosis of heparin-induced thrombocytopenia (HIT) remains a major clinical concern. Immunoassays are highly sensitive, while confirmatory functional tests (based on heparin-dependent platelet activation) lack standardization. We evaluated the diagnostic performance of a functional flow cytometric assay (FCA) based on the detection of heparin-dependent platelet activation with an anti-p-selectin. A total of 288 patients were included (131 HIT-positive and 157 HIT-negative) with a HIT diagnosis established by expert opinion adjudication (EOA) considering clinical data and local laboratory results. The FCA was centrally performed in a single laboratory on platelet-rich plasma, using a very simple four-color fluorometer. The results were standardized according to the Heparin Platelet Activation (HEPLA) index. The serotonin release assay (SRA) was performed in the four French reference laboratories. Based on the final HIT diagnosis established by EOA, the sensitivity and specificity of the FCA were 88 and 95%, respectively, values very similar to those of the SRA (88 and 97%, respectively). This study showed that the FCA, based on easily implementable technology, may be routinely used as a reliable confirmatory test for HIT diagnosis.

## 1. Introduction

Heparin-induced thrombocytopenia (HIT) is an adverse drug reaction caused by platelet-activating antibodies that are produced as a result of heparin therapy. These antibodies, generally directed against the platelet factor 4/heparin (PF4-H) complex, activate platelets via the FcϒRII receptor. HIT diagnosis is suspected on the basis of the timing and kinetics of the fall in platelet count, as well as on clinical criteria. In approximately 50% of cases, venous and arterial thrombosis is observed at the time of HIT diagnosis [1].

As thrombotic complications are frequent in HIT patients, the rapid and reliable diagnosis of this condition, allowing an immediate switch to an alternative anticoagulant, is of great importance. Delaying this decision may be life-threatening for patients with HIT [2] while a switch to an alternative anticoagulant in non-HIT patients may be associated with a major risk of bleeding [3,4]. Currently, HIT diagnostic algorithms are based on clinical probability (evaluated according to the 4Ts score) and the results of a sensitive immunoassay detecting PF4-H antibodies to guide initial management, with subsequent confirmatory testing by a more specific functional assay for patients with a positive immunoassay [5,6]. However, widely available sensitive immunoassays lack specificity, especially in patients having undergone cardiopulmonary bypass [7], while more specific functional assays, such as the heparin-induced platelet activation assay (HIPA), or platelet aggregation assays, such as light transmission aggregometry (LTA) or the 14C serotonin release assay (SRA), are not widely available owing to technical limitations and to the requirement of specialized resources. The laboratory diagnostic work-up for HIT can therefore take several days. The evolution of platelet count after heparin withdrawal is one of the criteria used to evaluate the probability of HIT. Taking together all the clinical criteria, in conjunction with laboratory results, can lead to a quite reliable HIT diagnosis, especially when evaluated by experts [8].

Like some other functional assays, such as the SRA, the flow cytometric assay (FCA) evaluates the capacity of the patient’s HIT antibodies to induce normal donor platelet activation in the presence of heparin. Both assays detect exocytosis upon platelet activation, but whereas the SRA measures the release of soluble exogenous radiolabeled serotonin from dense granules, the FCA detects exposure of the α granule membrane protein, p-selectin, translocated to the platelet surface upon activation [9]. The high sensitivity and specificity of the SRA have led to its being considered as the “gold standard” for HIT diagnosis. However, it is time-consuming, is generally performed only on a limited number of days and requires the preparation of washed platelets, a procedure necessitating considerable expertise to achieve reproducible and reliable assay results. The other main drawback of the SRA is the need for radioactive products, restricting use of this assay to only a few laboratories. At present, HIT diagnosis is still in need of a fast, standardized and nevertheless highly specific test that could be easily implemented in all hemostasis laboratories. Unlike the SRA, the FCA is not performed on washed platelets but on platelet-rich plasma (PRP) like Platelet Aggregation Tests (PAT). The reported performance of the FCA with P-selectin expression evaluated on small numbers of patients varies from one study to another depending on the method used [9,10,11].

The aim of this study was to evaluate the performance of the FCA with reference to the HIT diagnosis established on the basis of clinical expert opinion and to compare its sensitivity and specificity to those of the SRA.

This was an ancillary study performed in the context of the prospective observational “HIT score” study (registration no. NCT00748839) conducted in 31 tertiary hospitals (28 in France, 2 in Switzerland, and 1 in Belgium) under the auspices of the Groupe Français Hémostase et Thrombose (GFHT). The study was developed and coordinated by the Centre for Clinical Investigation (CIC 1408) of the Centre Hospitalier Universitaire, Saint-Etienne, France.

## 2. Materials and Methods

### 2.1. Design of the HIT Score Study

#### 2.1.1. Patients

All patients aged ≥18 years were eligible for inclusion if HIT was suspected on the grounds of newly developed thrombocytopenia or drop in platelet count, or venous or arterial thrombosis, during administration of UnFractionned Heparin (UFH), low molecular weight heparin (LMWH), or fondaparinux, and if an immunoassay for HIT antibodies was performed in a specialized hemostasis unit participating in the study. Patients were not included if the physicians responsible for their care: (i) considered there were insufficient clinical data prior to laboratory testing; (ii) considered the timing of thrombocytopenia to be unclear; (iii) detected a definite alternative cause of thrombocytopenia; (iv) could not ensure follow-up of the patient, in particular to assess whether platelet count normalized.

A total of 2280 patients were included in the HIT score study, which evaluated the clinical criteria associated with the diagnosis of HIT, between March 2009 and February 2013.

Data for each patient were recorded locally by the persons responsible for laboratory testing and pharmacovigilance, and the senior physician in charge of the patient, using a Case Report Form (CRF), study no. NCT00748839 (March 2009, Commission National Informatique et Liberté no. 908,138 and French Advisory Committee on the Processing of Information in the Field of Health Research no.08077) (see Figure S1).

#### 2.1.2. HIT Diagnosis

In the “HIT score” study, the HIT diagnosis was based on expert opinion adjudication (EOA). For each patient included HIT diagnosis was established by two independent experts (three in the event of discordance) on the basis of the electronic Case Report Form (eCRF), clinical and local laboratory data, including platelet count changes over time, results of laboratory testing for HIT antibodies (immunoassays and functional assays carried out as per routine care at each participating institution), and follow-up data until hospital discharge. Based on these data, the experts were able to classify HIT as certainly negative or certainly positive in 675 and 264 patients, respectively. No patient for whom the diagnosis was only considered as “possible HIT” was included in the FCA evaluation.

Platelet-poor plasma (PPP) was prepared at the time of HIT diagnosis by centrifugation of plasma samples at 2500× *g* for 15 min. Remaining plasma was stored at −80 °C for a second enzyme-linked immunosorbent assay (ELISA) (centrally performed) and performance of a SRA was entrusted to four different laboratories. A total of 200 HIT-positive patients out of 264, and 200 HIT-negative patients out of 675 were randomly selected for assessment by FCA. Sufficient plasma remained to perform FCA, on platelets from two donors, for 131 out of 200 randomly selected HIT-positive patients and for 157 out of 200 randomly selected HIT-negative patients.

The technicians performing the different assays (ELISA, SRA, and FCA) were not aware of the HIT diagnosis results.

### 2.2. ELISA and SRA

The presence in the patient’s plasma of Immunoglobulin G (IgG) antibodies against PF4-heparin complexes was centrally evaluated by ELISA (Zymutest^®^, Hyphen Biomed, Neuville-sur-Oise, France), according to the manufacturer’s protocol. The wells in the Micro Elisa plate were coated with unfractionated heparin, biologically available and supplemented with a platelet lysate providing PF4 molecules.

The diluted plasma was introduced in the microwells in duplicate. When present, heparin-dependent antibodies bound heparin PF4 complexes. Following a washing step, IgG bound antibodies were revealed with the immunoconjugate (anti-human IgG goat polyclonal antibody) (Fcγ specific)-peroxidase (HRP) conjugate. The Optical Density (OD) measured at a wavelength of 450 nm was directly proportional to the amount of heparin-dependent IgG antibodies. An OD of 0.3 was defined as the cut-off value by the manufacturer.

SRA was performed in four centers, as previously described [12]. The assay was defined as positive if a serotonin release >20% was measured at low heparin concentration (0.1 or 0.5 IU/mL depending on the center) but not at high heparin concentration (10 or 100 IU/mL depending on the center). The assay was considered as indeterminate if a serotonin release >20% was measured in the presence of buffer alone or if the serotonin release was between 20 and 30% in the presence of a low heparin concentration. All SRA were performed on platelets from two healthy donors (selected for their platelet sensitivity to heparin-dependent antibodies). The patient was considered SRA-positive if the result was positive with platelets from at least one donor.

### 2.3. Flow Cytometric Assay (FCA)

The FCA was performed as described by Tomer et al. [9]. This FCA is based on the capacity of HIT antibodies to activate platelets in the presence of heparin.

Whole blood from unmedicated healthy volunteers was drawn into citrated vacuum tubes and centrifuged at 200× *g* for 15 min at room temperature to obtain PRP that was used for testing within 3 h after blood collection. The corresponding PPP obtained by centrifugation at 2500× *g* for 15 min, were used as negative controls. Frozen patient plasma and HIT positive control plasma were thawed 5 min at 37 °C.

Unfractionated porcine heparin sodium (5000 IU/mL) was obtained from Sanofi (Sanofi, Ploërmel, France), a mixture with 3 IU/mL of UFH being prepared for the low concentration and a mixture with 1000 IU/mL of UFH for the high concentration. Phosphate-buffered saline (PBS) was obtained from Dutscher (Dutscher, Bernolsheim, France). Thrombin Receptor Agonist Peptide (TRAP) was purchased from VWR (VWR, Strasbourg, France) and reconstituted with 500 µL of sterilized water for a concentration of 1 mg/mL. Phycoerythrin (PE)-conjugated monoclonal mouse anti-human CD41 antibody (mAb) directed against platelet glycoprotein IIb/IIIa Clone 5B12, was purchased from Dako (Agilent Technologies, Les Ulis, France) and fluorescein isothiocyanate (FITC)-conjugated mouse anti-human CD62 mAb directed against activated platelet P-selectin (clone AK6), from Serotec (Bio-rad, Oxfordshire, UK).

The antibody mix was prepared as follow: 42 µL of PBS + 1 µL of PE-conjugated anti-CD41a (final concentration 1.5 µg/mL) and 2 µL of FITC-conjugated anti-CD62 mAb (final concentration 2 µg/mL).

First step: preparation of different mixtures and first incubation:

The level of spontaneous platelet activation was assessed on a mixture of donor PRP (10 µL) + donor PPP (10 µL) + PBS (30 µL) as a negative control (PBS Ctl−).

Donor platelet activity was assessed on a mixture of donor PRP (10 µL) + donor PPP (10 µL) + PBS (30 µL) + TRAP (8 µL; final concentration 100 µg/mL, added 5 min before the end of the first incubation) as a positive TRAP control (TRAP Ctl+).

The absence of control platelet activation in the presence of UFH itself was checked on a mixture of donor PRP (10 µL) + donor PPP (10 µL) + PBS + UFH 0.3 IU/mL (final concentration) as a negative Heparin control (HEP Ctl−).

Platelet sensitivity to HIT antibodies was verified using HIT-positive control plasma (HIT Ctl+) obtained from patients with confirmed HIT.

Donor PRP (10 µL) added to HIT-positive PPP (10 µL) was mixed with either UFH 0.3 IU/mL (final concentration) or UFH 100 IU/mL (final concentration) and/or PBS.

To detect the presence of HIT antibodies in patient plasma, we prepared different mixtures of donor PRP (10 µL) added to patient PPP (10 µL) with either UFH 0.3 IU/mL (final concentration) or UFH 100 IU/mL (final concentration) and/or PBS.

All the mixtures prepared were incubated on an orbital shaker (at 50 rpm) for 1 h at room temperature. This gentle shaking is very important.

Second step: incubation with antibodies

A total of 5 µL microliters of PBS Ctl−, TRAP Ctl+, HEP Ctl−, HIT CTl+, and the different mixtures of donor PRP + patient PPP were incubated for 15 min at room temperature with 45 µL of the antibody mix.

Platelet activation was stopped by adding PBS (450 µL), the samples then being immediately analyzed by flow cytometry.

Third step: analysis of activated platelet

**Figure 1:**
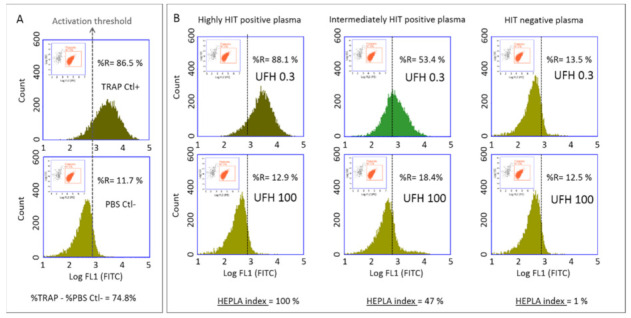
Dot plot: Log SSC (Side Scatter) versus Log FL2 (Filter 2) (in orange). A gate was drawn around CD41+ events, delineating the platelet population. FL1 Histogram: count versus log FL1 (in green). The gate CD41+ was applied to analyze FL1. ‘%R’ represents the percentage of CD-62p positive events. (**A**) Upper panels, TRAP-activated platelets (TRAP Ctl+); lower panels, resting platelets (PBS Ctl−). A total of 10,000 platelets (CD41+ events) were analyzed in each run and the percentage of activated platelets (CD62p positive events) was evaluated (FL1 histogram). For each series, a cursor indicating the activation threshold was placed at the intersection of the FL1 histograms of the negative control (PBS Ctl−) and the positive control (TRAP Ctl+) and the cut-off was determined. In this example, with resting platelets (PBS Ctl−), we observed 11.7% CD62p-positive events corresponding to spontaneous platelet activation and electronic noise. After platelet activation with TRAP (TRAP Ctl+), we observed 86.5% CD62p-positive events corresponding to maximal platelet activation. (**B**) Left panels: Platelets activated with a highly HIT positive plasma. We observed 88.1% CD62p-positive events with 0.3 IU/mL. UFH (upper panel) and 12.9% CD62p-positive events with 100 IU/mL UFH (lower panel). The HEPLA index was 100% Middle panels: Platelets activated with intermediately HIT positive plasma. We observed 53.4% CD62p-positive events with 0.3 IU/mL UFH (upper panel) and 18.4% CD62p-positive events with 100 IU/mL UFH (lower panel). The HEPLA index was 47%. Right panels: Platelets incubated with HIT negative plasma. We observed 13.5% CD62p-positive events with 0.3 IU/mL UFH (upper panel) and 12.5% with 100 IU/mL UFH (lower panel). The HEPLA index was 1%. TRAP: Thrombin Receptor Agonist Peptide, PBS: Phosphate-buffered saline, HIT: Heparin-induced thrombocytopenia, UFH: Unfractioned heparin, HEPLA: Heparin Platelet Activation.

Platelet activation was analyzed by standard two-color flow cytometry using a BD Accuri C6 flow cytometer (Becton Dickinson, San Jose, CA, USA) equipped with two light scatter detectors and four fluorescence detectors. A daily internal quality control of the instrument was performed using Spherotech 8-peak validation beads (Becton Dickinson, San Jose, CA, USA) according to the manufacturer’s protocol. Fluorescence compensation was applied to correct emission spectrum overlap between FITC (FL1 filter, 17%) and PE (FL2 filter, 2%), using single-color-labeled preparations. Logarithmic side scatter (SSC) versus Log FL2 (PE-labeled anti-CD41a antibody) gating enabled differentiation between the platelet population (CD41a+) and cell debris (CD41a−). Activated platelets were identified by FITC-labeled anti-CD62p mAb. Platelets were analyzed according to the histogram defined by counts versus logarithmic FITC fluorescence (FL1) (Figure 1). A total of 10,000 platelets (CD41+ events) were analyzed in each sample and the percentage of activated platelets (CD62p positive events) was evaluated (FL1 histogram). For each series, a cursor indicating the activation threshold was placed at the intersection of the FL1 histograms of the negative control (PBS Ctl−) and the positive control (TRAP Ctl+) (Figure 1A). This set-up enabled determination of the percentage of activated platelets in the presence of patient plasma (to the right of the activation threshold) in the presence of either 0.3 IU/mL or 100 IU/mL UFH (Figure 1B).

### 2.4. Standardized Expression of the Results

Platelet activation is expressed as the percentage of the activated platelets (identified as CD62+) within the platelet population (identified as CD41+). This platelet activation was evaluated by measuring the expression of CD62p under four different conditions: with 0.3 IU/mL UFH (H 0.3) and 100 IU/mL UFH (H 100) in the presence of patient PPP, and with TRAP and PBS in the presence of control PPP. We standardized the expression of platelet activation according to the Heparin Platelet Activation (HEPLA) index that takes into account platelet activation measured under these four conditions. The HEPLA index is defined as follows:

HEPLA index= [% H 0.3 − % H 100]/[% TRAP Ctl+ − % PBS Ctl−] × 100(1)

If residual heparin is present in the patient’s PPP, basal platelet activation may be observed before the addition of heparin to the mixture. As no platelet activation should be observed with H 100, we used H 100 rather than H 0 for HEPLA calculation, allowing a reliable conclusion to be drawn even in the presence of residual heparin in the plasma.

### 2.5. Determination of HEPLA Index Cut-Off

The mean HEPLA index measured in presence of PPP and PRP from 28 healthy blood donors was 3.8 ± 6.3% (95% CI 2.6% to 5.2). The HEPLA index cut-off for platelet activation defined by the mean + 2 SD was 16.5.

### 2.6. Validation Rules

Each series was technically validated on the basis of a positive TRAP Ctl+, confirming that the platelets were functional, and a HEPLA index above 16.5% for the HIT Ctl+, confirming that the platelets were sensitive to HIT antibodies. A patient’s plasma was considered HIT-positive if the HEPLA index exceeded 16.5% with platelets from at least one of the two donors.

### 2.7. Statistical Analysis

Flow cytometric data were analyzed by means of BD software. The coefficient of variation (CV), the mean and the standard deviation (SD) were calculated using Graph pad Prism. The sensitivity, specificity and confidence Interval (CI) were calculated using Graph pad Prism.

The accuracy of the FCA in discriminating HIT-negative patients from HIT-positive patients was assessed according to the receiver operating characteristic (ROC) plot, i.e., the graph of sensitivity versus (1 minus specificity), as the threshold for the assay varies over all possible sensitivity and specificity values. The area under the ROC curve (AUC) was considered as a summary index of accuracy. The ROC curve was calculated using Graph pad Prism.

The FCA and SRA results were compared by calculating the percentage of agreement between the two assays (based on the Kappa test ratio) using Graph pad Prism.

The comparison of the FCA, SRA, and EOA was represented by a Venn diagram using “R” software with the package “VennDiagram”.

## 3. Results

### 3.1. Performance of the FCA for HIT Diagnosis Based On EOA

The intra-assay coefficient of variation (CV) of the FCA, calculated on 11 measurements of HIT Ctl+, was 5.7%. The inter-assay CV of the FCA, obtained by testing the same HIT Ctl+ in 17 different series, using platelets from 17 different donors, was 23.2% (data not shown).

The mean percentage of spontaneous platelet activation (PBS Ctl−) observed in the different series was 12.9 ± 7.8% (95% confidence interval (CI) 11.3 to 14.5%). The mean percentage of maximal platelet activation (TRAP Ctl+) observed in the different series was 78.9 ± 7.5% (95% CI 77.4 to 80.4%) (*n* = 96).

**Figure 2:**
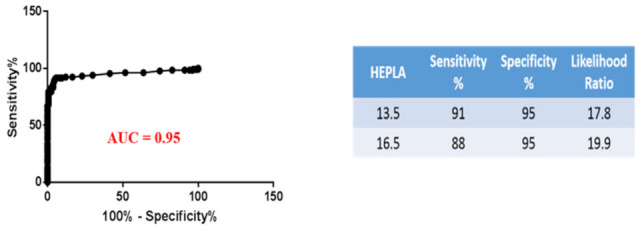
Diagnostic performance of FCA assessed by receiver operating characteristic (ROC) analysis of the HEPLA index (*n* = 288), yielding an area under the curve of 0.95. The diagnosis of HIT was based on EOA as described in the “HIT diagnosis” section. FCA: Functional Cytometric assay, ROC: receiver operating characteristic, EOA: expert opinion adjudication.

Agreement between the HEPLA index and the EOA HIT diagnosis in 288 patients was assessed by ROC analysis: the AUC was 0.95 (95% CI: 0.920–0.980, *p* < 0.0001). The HEPLA Index cutoff value of 13.5 indicated by the ROC analysis gave the best sensitivity and specificity. However, the HEPLA index cut-off value of 16.5, calculated as the mean + 2 SD, gave the best likelihood ratio (19.9) (Figure 2).

When 16.5 was considered as the HEPLA index cut-off value, the sensitivity and specificity of the FCA were 88 and 95%, respectively. When the ELISA IgG results were taken into account in conjunction with the FCA results, the sensitivity was almost the same (87%), but the specificity increased to 98%.

A disagreement between the FCA results and the EOA HIT diagnosis was observed in 8% of patients, with 15 false-negative and 7 false-positive results.

Among the 15 false-negative FCA results: ELISA IgG OD was <0.3 in two cases, ELISA IgG OD was < 1 in five other cases and ELISA IgG OD was between 1.2 and 2.8 for eight cases. Among the 7 false-positive FCA results, ELISA IgG OD was >1 in two cases. Details of the disagreements between the FCA results and the EOA HIT diagnosis are provided in Table 1.

### 3.2. Performance of the SRA for HIT Diagnosis Based On EOA

Based on the final HIT diagnosis established by EOA, the sensitivity and specificity of the SRA were 88 and 97%, respectively. SRA results considered as doubtful in 13 cases were not taken into account for calculation of the sensitivity and specificity. SRA results were considered as doubtful on the grounds of a weak (20 to 30%) serotonin release in two cases and because of a spontaneous (without heparin) serotonin release in 11 cases with a persistent serotonin release at high heparin concentration in 5 out of 11 cases. When IgG ELISA results were coupled with SRA results, the sensitivity and specificity of the SRA moved to 86 and 99%, respectively.

We observed discrepancies between the SRA results and the EOA HIT diagnosis for 6.5% of patients (15 false-negative and 4 false-positive results). Among the 15 false-negative SRA results: no IgG were detected using ELISA for two patients, ELISA IgG OD was <1 for four patients and ELISA IgG OD was between 1.2 and 2.6 for nine patients. Among the four false-positive SRA results, ELISA IgG OD was >1.0 in only one case. Details of the discrepancies between the SRA results and the experts’ opinions are given in Table 2.

### 3.3. Comparison of the FCA with the SRA and Analysis of Discordant Results

The sensitivity and specificity of the FCA were identical to those of the SRA. The IgG ELISA slightly increased the specificity of FCA and SRA (Table 3).

**Figure 3:**
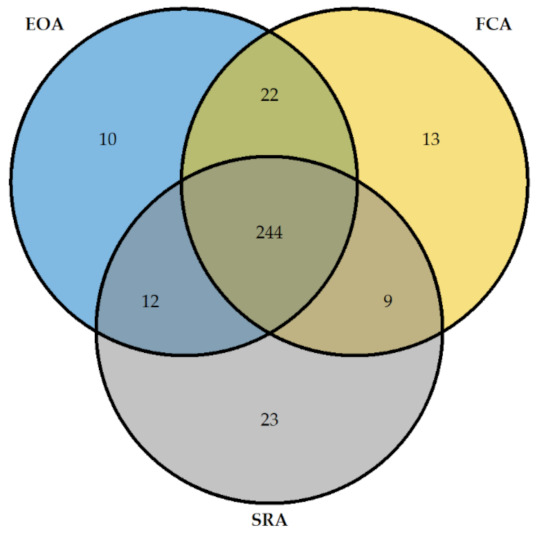
Venn diagram for EOA, FCA, and SRA decisions on 288 patients. This Venn diagram represents the positive and negative decisions. For the SRA, the indeterminate results are not included. Each circle represents one classifier (blue: EOA, yellow: FCA and grey: SRA). The number of agreements between two classifiers is noted in the intersection of two circles, and the number of agreements between three classifiers is noted in the intersection of three circles. The number of decisions taken on the basis of only one classifier is noted in the non-superposed part of the circle. EOA: Expert opinion adjudication, FCA: Functional cytometric assay, SRA: Serotonin release assay, HIT: Heparin-induced thrombocytopenia.

The results given by FCA, SRA, and EOA HIT diagnosis were superposable for 244 patients (Figure 3).

The percentage agreement between the FCA and the SRA was 92% and the Kappa test ratio was 0.820 (95% CI: 0.769–0.901) (Table 4) indicating a good strength of agreement between the two assays.

However, there were some discordances between the FCA and SRA results for 22 patients as summarized in Table 5.

Among the 13 patients with positive FCA results and negative SRA results, 8 had been diagnosed as HIT-positive by EOA, with an ELISA IgG OD >1 in 7 cases. The remaining five patients with positive FCA and negative SRA results had been classified as HIT-negative by EOA, ELISA IgG OD being >1 in only one of these patients. Among the nine patients with negative FCA and positive SRA results, seven had been diagnosed as HIT-positive by EOA, ELISA IgG OD being >1 in five of these patients. In contrast, neither of the two patients with negative FCA results and positive SRA results classified as HIT-negative by EOA had a positive IgG ELISA. The values of the HEPLA index (FCA) and serotonin release (%) (SRA) for patients with the false negative and false positive results (according to EOA HIT diagnosis) are presented in Table 6 and Table 7.

### 3.4. Indeterminate Results of FCA and SRA

No FCA result was considered as doubtful. In total, 13 SRA results were considered as doubtful either because of a low serotonin release (20 to 30%) in 2 cases (with a HEPLA index of 0 and 5%, respectively) or because of a spontaneous serotonin release measured in the presence of buffer only in 11 cases. For 5 out of these 11 doubtful results, serotonin release was still observed in the presence of a high heparin concentration. Of these five results, the two associated with a very low HEPLA index were probably related to spontaneous donor platelet activation due to the platelet washing procedure while the three associated with a high HEPLA index were probably related to the presence of some activating factor (such as thrombin) in the patient’s plasma. For the remaining 6 out of the 11 doubtful results, no serotonin release persisted in the presence of a high heparin concentration; in the four cases with a very high HEPLA index and a positive HIT diagnosis, the spontaneous serotonin release was probably related to the presence of residual heparin in the patient’s plasma; in contrast, in the two cases with a very low HEPLA index and a negative HIT diagnosis, the spontaneous serotonin release was rather related to the platelet washing procedure.

## 4. Discussion

In this study, we used a standard flow cytometry apparatus equipped with only two light scatter detectors and four fluorescence detectors, considering that the simpler the equipment, the easier it is to select the settings. Comparing the FCA and SRA results with the HIT diagnoses established by EOA, the FCA displayed both high sensitivity and specificity (88 and 95%, respectively), comparable to those of the SRA. These results were obtained on an unprecedentedly large number of definitively HIT-positive samples (with a nearly equal number of definitively HIT-negative samples) ensuring high reliability of the results of both assays.

As in all other functional tests, performance of the FCA depends on the sensitivity of the donor’s platelets to the patient’s HIT antibodies. This explains why the repeatability of this assay (intra-assay CV: 5.7%) was much better than its reproducibility (inter-assay CV: 23%), the variation in HIT-positive control values between the different series being related to differences in the sensitivity of the control platelets to HIT antibodies (different control platelets being used in successive series). However, the platelet activation achieved with the HIT Ctl+ plasma included in each series confirmed that the platelets were sensitive to HIT antibodies (the condition for series validation). The HEPLA index takes into account platelet reactivity (assessed by the TRAP Ctl+) and may be considered as a standardized expression of the results.

Despite the very good agreement between the FCA or SRA results and the EOA HIT diagnosis, we still found some discordance within both assays. Of the 15 false-negative FCA results and the 15 false-negative SRA results, four in each case were observed in patient plasmas containing a low titer of anti-PF4-IgG antibodies (OD: 0.5–0.9), indicating a lower sensitivity of the FCA and SRA compared to the IgG ELISA [13]. Among the 15 false-negative FCA results, 8 were observed in patient plasmas with a high titer of anti-PF4-H antibodies (OD: 1.2–2.8). In five of these eight cases, the SRA results were positive, the greater sensitivity of the SRA in these cases probably being due to the use of washed platelets. Of the 15 false-negative SRA results, 9 were obtained in patient plasmas containing a high titer of anti-PF4 Ig G antibodies (OD: 1.2–2.6), the positive FCA results obtained in 8 of these cases reflecting a lower sensitivity of the SRA possibly due to low levels of PF4 in the reaction mixture as already described [14,15]. At the time this study was performed, addition of PF4 to the reaction mixture was not a usual procedure in any centralized laboratory, so the rate of false-negative SRA results related to low PF4 levels cannot be estimated.

The sensitivity of the FCA was identical to that of the SRA, which was not as good under our study conditions as when the assay is centrally performed in a single specialized laboratory [16]. Although we made sure that the four centers responsible for performing the centralized SRA in our study used the same previously reported method [12], a 4.5% rate of indeterminate reaction patterns was observed. As the SRA necessitates the washing of platelets, technically a very demanding procedure, some differences in the performance of this assay might be expected between laboratories [17]. Indeed, as previously reported, the key challenge with the SRA is not so much the measurement of serotonin release per se, but rather the handling and preparation of the suspended platelets (with the inherent risk of spontaneous platelet activation), which can differ greatly between laboratories. It is therefore understandable that the performance characteristics (in terms of sensitivity and specificity) reported by one laboratory do not necessarily apply to others [16]. As there is no requirement for washing platelets when using the FCA, we may expect that the quality of the results obtained with this assay would be unlikely to vary between different centers. Moreover, the incidence of indeterminate reaction patterns observed for the SRA (up to 10% in routine practice), could have been reduced by including some additional procedures, such as heat-inactivation of patient sera, or the use of sample dilutions, as already suggested [16]; however, neither of these procedures was routinely implemented at the time this study was performed. If residual heparin is present in the patient’s plasma, basal platelet activation may be observed before addition of heparin to the mixture. In the case of HIT, no platelet activation should be observed in the presence of a high UFH concentration. The point corresponding to inclusion of 100 IU UFH is more relevant for HEPLA index calculation than that determined in the absence of added heparin, allowing for FCA (but not for SRA) a reliable conclusion even in the presence of residual heparin in the plasma.

Specificity is also an important aspect of functional assay performance. In our study, both the FCA and the SRA displayed a good specificity (95 and 97%, respectively). When the FCA and SRA results were considered in conjunction with those of the IgG ELISA, the specificities of both assays were further slightly improved without a major change in sensitivity, as already demonstrated for the association of SRA results with those of the IgG ELISA by Warkentin et al. [18]. However, it is also conceivable that some of the negative ELISA results obtained on plasmas giving positive results with the SRA and/or the FCA are related to the presence of IgG antibodies directed against other target chemokines, such as IL 8 or NAP2 [19].

The performances of the FCA and SRA might have been underestimated in our study. In particular, for seven patients, the experts’ diagnostic classification might have been mistaken. Among the false-negative FCA and SRA results, four were observed in patient plasma samples containing no or a very low titer of anti-PF4-H IgG antibodies (OD: 0.1–0.6) (Table 1 and Table 2). Similarly, 2 out of 7 false-positive FCA results were obtained in plasma samples with a high IgG OD in the ELISA (Table 1) and 1 out of 4 false-positive SRA results were obtained in plasma samples with a high IgG OD in the ELISA (Table 2). As no HIT diagnostic assay can be considered as completely reliable and as expert opinion can always be disputed, the use of a multiparametric adjudication has been proposed for routine practice [3,8].

In four other studies where annexin V was used as the marker of platelet activation, the FCA evaluated versus the SRA or heparin-induced platelet activation (HIPA) or clinical diagnosis [9,20,21,22] showed very good sensitivity (varying from 88 to 95%) and specificity (varying from 96 to 100%). In the studies where P selectin was used as platelet activation marker, the performances of the FCA could be considered as closely to similar [23] except in one study [11]. Evaluation of the commercial FCA Emo-test HIT confirm^®^ showed lower performances when this assay was performed with only one unselected platelet control, under different experimental conditions (donor PRP incubated with serum for only 30 min (instead of 60 min) without gentle shaking) and using a lower HEPLA index cut-off value, namely 13%, suggesting possible explanations for the lower sensitivity (69.7%) and lower specificity (75%) reported [11].

Under our experimental conditions, the FCA displayed good sensitivity and specificity in accordance with the results of other studies [23]. Moreover, the absence of any requirement for washing platelets when using the FCA makes this assay much less demanding than the SRA and therefore widely available in most laboratories even in tertiary care hospitals [21]. As the SRA is very time-consuming (necessitating one day for each series and for this reason performed only every one or two weeks), HIT diagnosis based on the SRA is always delayed compared to HIT diagnosis using the FCA.

Limitations of our study are that the SRA was performed in four different central laboratories, which could have impacted its accuracy, and that the platelets used for the FCA and the SRA were not derived from the same donors.

## 5. Conclusions

Even though the inter-laboratory variability in FCA performance remains to be evaluated in a multicenter study, the results reported here support our proposal of the FCA as a rapid and reliable functional test for HIT diagnosis. The good performance of FCA nevertheless relies on the strict respect of the analytical conditions described in this study and on our HEPLA index calculation.

Use of the FCA in conjunction with an immunoassay detecting anti-PF4-H IgG antibodies could greatly increase confidence in switching to a non-heparin anticoagulant.

## Figures and Tables

**Table 1:** False-negative and false-positive FCA results (based on EOA HIT diagnosis).

	EOA HIT Diagnosis	FCA	SRA	Zymutest HIA IgG Cut-Off 0.3	IgG OD
1	+	−	−	−	0.1
2	+	−	−	−	0.2
3	+	−	−	+	0.5
4	+	−	−	+	0.6
5	+	−	−	+	0.7
6	+	−	−	+	0.9
7	+	−	−	+	1.2
8	+	−	−	+	2.4
9	+	−	+	+	0.7
10	+	−	+	+	1.2
11	+	−	+	+	1.4
12	+	−	+	+	2.1
13	+	−	+	+	2.5
14	+	−	+	+	2.8
15	+	−	Non-specific platelet activation	+	2.1
16	−	+	−	−	0.1
17	−	+	−	−	0.1
18	−	+	−	−	0.1
19	−	+	−	−	0.1
20	−	+	−	+	2.5
21	−	+	+	+	0.7
22	−	+	+	+	1.7

EOA: Expert opinion adjudication, HIT: Heparin-induced thrombocytopenia, FCA: Functional cytometric assay, SRA: Serotonin release assay, OD: Optical density.

**Table 2:** False-negative and false-positive SRA results.

	EOA HIT Diagnosis	SRA	FCA	Zymutest HIT IgG Cut-Off 0.3	IgG OD
1	+	−	−	−	0.1
2	+	−	−	−	0.1
3	+	−	−	+	0.5
4	+	−	−	+	0.6
5	+	−	+	+	0.7
6	+	−	−	+	0.9
7	+	−	−	+	1.2
8	+	−	+	+	1.7
9	+	−	+	+	1.8
10	+	−	+	+	2
11	+	−	+	+	2.2
12	+	−	+	+	2.4
13	+	−	+	+	2.4
14	+	−	+	+	2.4
15	+	−	+	+	2.6
18	−	+	−	−	0
19	−	+	−	−	0
20	−	+	+	+	0.7
21	−	+	+	+	1.7

EOA: Expert opinion adjudication, HIT: Heparin-induced thrombocytopenia, SRA: Serotonin release Assay, FCA: Functional cytometric assay, OD: Optical density.

**Table 3:** Sensitivity and specificity of the FCA and SRA alone and in conjunction with IgG ELISA for 288 patients (131 HIT+ and 157 HIT−).

Assay	Sensitivity	Specificity
FCA	88	95
SRA	88	97
FCA + IgG ELISA	87	98
SRA + IgG ELISA	86	99

FCA: Flow cytometric Assay, SRA: Serotonin release Assay, HIT: Heparin-induced thrombocytopenia.

**Table 4:** Agreement between the FCA and SRA results evaluated by the Kappa test ratio. SRA indeterminate results were deleted for this analysis.

Patients with Suspected HIT (*n* = 275)		SRA	
		Positive	Negative
**FCA**	Positive	103	**13**
	Negative	**9**	150
**Number of observed agreements = 253 (92%)**			
**Kappa test ratio: 0.820; 95% CI: 0.769–0.901**			

FCA: Flow cytometric assay, SRA: Serotonin release assay, HIT: Heparin-induced thrombocytopenia.

**Table 5:** Discordances between the FCA and SRA results.

**FCA+/SRA−**			***n* = 13 Patients**			
**FCA**	**SRA**	**EOA HIT Diagnosis**	**Zymutest^®^ HIT IgG Cut** **-** **Off 0.3**	**IgG OD**	**ECC**	**TE**
+	−	+	+	0.7	Yes	Yes
+	−	+	+	1.7	Yes	No
+	−	+	+	1.8	No	No
+	−	+	+	2	No	No
+	−	+	+	2.2	No	No
+	−	+	+	2.4	Yes	No
+	−	+	+	2.4	Yes	Yes
+	−	+	+	2.6	Yes	No
+	−	−	+	2.5	Yes	No
+	−	−	−	0	No	No
+	−	−	−	0	No	No
+	−	−	−	0	Yes	No
+	−	−	−	0.1	No	No
**FCA−/SRA+**			***n* = 9 patients**			
−	+	+	+	0.7	Yes	Yes
−	+	+	+	0.7	Yes	No
−	+	+	+	1.2	Yes	No
−	+	+	+	1.4	Yes	No
−	+	+	+	2.1	Yes	No
−	+	+	+	2.5	No	Yes
−	+	+	+	2.8	Yes	No
−	+	−	−	0	No	No
−	+	−	−	0	No	No

FCA: Flow cytometric assay, SRA: Serotonin release assay, EOA: Expert opinion adjudication; HIT: Heparin-induced thrombocytopenia, OD: Optical density, ECC: ExtraCorporeal circulation, TE: Thrombotic event.

**Table 6:** Values of the HEPLA index (%) and serotonin release (%) for false negative results of FCA and/or SRA. The values of the false negative results are in bold.

HEPLA Index%(Cut-Off: 16.5%)	Serotonin Release%(Cut-Off: 20%)
**−38**	89
**−4**	46
**4**	29
**6**	23
**8**	26
**13**	54
**14**	75
**16**	54
32	**1**
70	**2**
93	**3**
54	**9**
66	**10**
86	**10**
50	**14**
30	**19**
**0**	**1**
**1**	**6**
**1**	**19**
**3**	**1**
**4**	**1**
**5**	**5**
**14**	**1**

FCA: Flow cytometric assay, SRA: Serotonin release assay.

**Table 7:** Values of the HEPLA index (%) and serotonin release (%) for false positive results of FCA and/or SRA. The values of the false positive results are in bold.

HEPLA Index%(Cut-Off: 16.5%)	Serotonin Release%(Cut-Off: 20%)
**17**	2
**23**	8
**33**	1
**33**	10
**61**	2
7	**36**
1	**41**
**28**	**30**
**33**	**87**

TFCA: Flow cytometric assay, SRA: Serotonin release assay.

## Data Availability

Not applicable.

## Supplementary Materials

The following are available online at https://www.mdpi.com/2227-9059/9/4/332/s1, Figure S1: Case Report Form of HIT score study.
