# Supplementary material for: Functional Flow Cytometric Assay for Reliable and Convenient Heparin-Induced Thrombocytopenia Diagnosis in Daily Practice
Source: Biomedicines. 2021 Mar 25;9(4):332. doi: 10.3390/biomedicines9040332 (PMC8064483; doi:10.3390/biomedicines9040332)
Supplement: Supplementary file 1 [file biomedicines-09-00332-s001.pdf]

Figure S1: Case Report Form

Heparin-Induced Thrombocytopenia:
Development and Validation
# of a Predictive Clinical Score

Patient : |__|__|__|

N° Patient : |__|__|__|

Inclusion criteria	YES NO
Age ≥ 18 years :	☐1 ☐2
Patient with suspected HIT requiring an ELISA assay:	☐1 ☐2
Possible follow-up of 4 days after ELISA test performance:	☐1 ☐2

↳ in the case of one negative response, do not include the patient

Patient data	
Year of birth:	|__|__|__|__|
Se:	
Male:	☐1
Female:	☐2

Date of HIT suspicion = D0 *(DDMMYY)*	|__|__||__|__||__|__|
This date could not be the day of test performance	

Past History and Current Context

Past History	YES	NO
History of myocardial infarct:	☐1	☐2
History of stroke:	☐1	☐2
History of venous thromboembolism:	☐1	☐2
History of vascular surgery:	☐1	☐2
History of HIT:	☐1	☐2

Current Context	YES	NO
Active cancer..	☐1	☐2
Peripheral arterial disease	☐1	☐
Pregnancy or post partum	☐1	☐2
Autoimmune disease	☐1	☐2
Sepsis	☐1	☐2
DIC..	☐1	☐2
Aortic Pump	☐1	☐2
Multiple blood transfusion	☐1	☐2
Major trauma..	☐1	☐2
Shock...	☐1	☐2
Inflammatory Syndrome	☐1	☐2
Hospitalisation in an intensive care unit..	☐1	☐2
Other :	☐1	☐1
....		
....		

Previous treatment with heparin	YES NO UNKOWN
Had the patient received a treatment with heparin, danaparoid or fondaparinux during the previous 3 months?	☐1 ☐2 ☐3
If yes 1. Type of treatment :	
1.1. UFH:	☐1 ☐2
1.2. LMWH:	☐1 ☐2
1.3. Fondaparinux:	☐1 ☐2
1.4. Danaparoid:	☐1 ☐2
1.5. Therapeutic dose:	☐1 ☐2
1.6. Prophylactic dose:	☐1 ☐2
2. Route of administration:	☐1 ☐2
2.1. Intravenous:	
2.2. Subcutaneous:	☐1 ☐2
2.3. Central intravenous catheter:	☐1 ☐2
2.4. Cardiopulmonary bypass:	☐1 ☐2
2.5. Dialysis circuit:	
	☐1 ☐2
	☐1 ☐2
	YES NO UNKNOWN
Had the patient received a treatment with heparin, danaparoid or fondaparinux later than within the previous 3 months?	☐1 ☐2 ☐3
If yes 1. Type of treatment :	
1.1. UFH:	☐1 ☐2
1.2. LMWH:	☐1 ☐2
1.3. Fondaparinux:	☐1 ☐2
1.4. Danaparoid:	☐1 ☐2
1.5. Therapeutic dose:	☐1 ☐2
1.6. Prophylactic dose:	☐1 ☐2
2. Route of administration:	
2.1. Intravenous:	
2.2. Subcutaneous:	☐1 ☐2
2.3. Central intravenous catheter:	☐1 ☐2
2.4. Cardiopulmonary bypass:	☐1 ☐2
2.5. Dialysis catheter:	

	☐1 ☐2<br>☐1 ☐2

Current Episode of HIT Suspicion

Current Treatment	☐ YES ☐ NO ☐ UNKNOWN
Type of treatment :<br>1. UFH: ....<br>2. LMWH: ....<br>3. Fondaparinux: ....<br>4. UFH and then LMWH: ....<br>5. LMWH and then UFH: ....<br>6. UFH and then fondaparinux: ....<br>7 .Fondaparinux and then UFH: ....<br>8. LMWH and then fondaparinux: ....<br>9. Fondaparinux and then LMWH:.......................<br><br>10. Therapeutic doses: ....<br>11. Prophylactic doses : ....<br>12. Prophylactic and then therapeutic doses: ......<br>13. Therapeutic and then prophylactic doses: .....<br>Route of administration no. 1 :<br>1. Intravenous: ....<br>2. Subcutaneous: ....<br>3. Central intravenous catheter....................................<br>4. Cardiopulmonary bypass ....<br>5. Dialysis catheter: ....<br>Route of administration no. 2 :<br>1. Intravenous: ....<br>2. Sub cutaneous: ....<br>3. Central intravenous catheter....................................<br>4. Cardiopulmonary bypass ....<br>5. Dialysis catheter:...<br><br>Had the «heparin» treatment been discontinued for at least 24 hours?	☐1 ☐2<br>☐1 ☐2<br>☐1 ☐2<br>☐1 ☐2<br>☐1 ☐2<br>☐1 ☐<br>☐1 ☐2<br>☐1 ☐2<br>☐1 ☐2<br><br>☐1 ☐2<br>☐1 ☐2<br>☐1 ☐2<br>☐1 ☐2<br><br>☐1 ☐2<br>☐1 ☐2<br>☐1 ☐2<br>☐1 ☐2<br><br>☐1 ☐2

If Yes :	☐1 ☐2
1. from 1 to 2 days :..	☐1 ☐2
2. from 2 to 3 days :..	☐1 ☐2
3. from 3 à 7 days :	☐1 ☐2
	☐1 ☐2
	☐1 ☐2
	☐1 ☐2
	☐1 ☐2
	☐1 ☐2

Current indication for the «heparin» treatment	YES	NO
Therapeutic doses	☐1	☐2
If Yes: Indication :		
1. Venous thromboembolism...	☐1	☐2
2. Cerebral venous thrombosis	☐1	☐2
3. Portal vein thrombosis	☐1	☐2
4. Other site of venous thrombosis	☐1	☐2
5. Acute coronary syndrome	☐1	☐2
6. Arrhythmia	☐1	☐2
7. Stroke	☐1	☐2
8. Valve prosthesis..	☐1	☐2
9. Arterial thrombosis of lower limbs...	☐1	☐2
10. Other site of arterial thrombosis..	☐1	☐2

11. Post-cardiac surgery (CPB) .... Date of CPB __/__/__...	☐1 ☐2
12 Other :...	☐1 ☐2
**Prophylactic:**	☐1 ☐2
<u>If Yes:</u> Indication :	
1. Medical	☐1 ☐2
2. Post-orthopedic surgery..	☐1 ☐2
3. Post-cardiac surgery (CPB)	☐1 ☐2
Date of CBP __/__/__	
4. Post-peripheral vascular surgery..	☐1 ☐2
5. Post-neoplastic surgery	☐1 ☐2
6. Other post-surgery	☐1 ☐2
7. Sequential hemofiltration	☐1 ☐2
8. Continuous hemofiltration	☐1 ☐2
9. Other..	☐1 ☐2

Platelet count before the «heparin» treatment	
Date of blood sample withdrawal:	|__|__| |__|__| |__|__|
1. Platelet count (Giga/L) :	|__|__|__|

Date of «heparin» therapy initiation	|__|__| |__|__| |__|__|
**Date of HIT suspicion**	|__|__| |__|__| |__|__|
**Number of days of « heparin » treatment**	|__|__|

Use of other drugs inducing thrombocytopenia	
Has the patient received other drugs inducing thrombocytopenia?	☐1 YES ☐2 NO
1. Antibiotic therapy:	☐1 YES ☐2 NO
If yes: 1.1. Quinolone	☐1 YES ☐2 NO
Start (DDMMYY)	|__|__| |__|__| |__|__|
End (DDMMYY)	|__|__| |__|__| |__|__|
	Not stopped |__|
1.2. ß lactam	☐1 YES ☐2 NO
Start (DDMMYY)	|__|__| |__|__| |__|__|
End (DDMMYY)	|__|__| |__|__| |__|__|
	Not stopped |__|
1.3. Vancomycin or teicoplanin.......	☐1 YES ☐2 NO
Start (DDMMYY)	|__|__| |__|__| |__|__|
End (DDMMYY)	|__|__| |__|__| |__|__|
	Not stopped |__|
1.4. Rifampicin or isoniazid	☐1 YES ☐2 NO
Start (DDMMYY)	|__|__| |__|__| |__|__|
End (DDMMYY)	|__|__| |__|__| |__|__|
	Not stopped |__|
1.5. Amphotericin or fluconazole....	☐1 YES ☐2 NO
Start (DDMMYY)	|__|__| |__|__| |__|__|
End (DDMMYY)	|__|__| |__|__| |__|__|
	Not stopped |__|

1.6. Other treatments	☐1 YES ☐2 NO
Start (DDMMYY)	|__|__| |__|__| |__|__|
End (DDMMYY)	|__|__| |__|__| |__|__|
	Not stopped |__|
2. Chemotherapy..	☐1 YES ☐2 NO
Start (DDMMYY)	|__|__| |__|__| |__|__|
End (DDMMYY)	☐☐☐☐☐☐☐☐
	Not stopped |__|
3. Anti-GPIIb IIIa	☐1 YES ☐2 NO
	|__|__| |__|__| |__|__|
Start (DDMMYY)	|__|__| |__|__| |__|__| Non stopped |__|
End (DDMMYY)	
4. Furosemide...	☐1 YES ☐2 NO
Start (DDMMYY)	|__|__| |__|__| |__|__|
End (DDMMYY)	|__|__| |__|__| |__|__|
	Not stopped |__|
5. Proton pump inhibitors	☐1 YES ☐2 NO
	|__|__| |__|__| |__|__|
Start (DDMMYY)	|__|__| |__|__| |__|__|
End (DDMMYY)	Not stopped |__|
6. Other treatments …………………………….	☐1 YES ☐2 NO
Start (DDMMYY)	|__|__| |__|__| |__|__|
End (DDMMYY)	|__|__| |__|__| |__|__|
	Not stopped |__|

Events occurring from heparin or fondaparinux therapy initiation to HIT suspicion	
Has the patient experienced one or more events?	☐1 YES ☐2 NO
If yes, 1. Thrombotic events:	☐1 YES ☐2 NO
1.2. New arterial thrombosis	☐1 YES ☐2 NO
- Site of this new arterial thrombosis	
_______1.2.1. Number of arterial thrombosis events:..	|__|
Date of the first event (DDMMYY)	|__|__| |__|__| |__|__|
Date of the second event (DDMMYY)	|__|__| |__|__| |__|__|
1.3. Pulmonary embolism	☐1 YES ☐2 NO
1.4. New venous Thrombosis	☐1 YES ☐2 NO
Date of the first event (DDMMYY)	|__|__| |__|__| |__|__|
Date of the second event (DDMMYY)	|__|__| |__|__| |__|__|
1.5. Extension of a previous venous thrombosis	☐1 YES ☐2 NO
1.6. Extension of a previous arterial thrombosis	☐1 YES ☐2 NO
1.7. Was the extension or the new thrombosis diagnosed by systematic ultrasonography (was the thrombotic event a non-symptomatic event ?)	☐1 YES ☐2 NO
2. Bleeding events:	☐1 YES ☐2 NO
If yes, 2.1.Requering blood transfusion	☐1 YES ☐2 NO
2.2. Requiring surgery...................................	☐1 YES ☐2 NO
Date of the bleeding(DDMMYY)	|__|__| |__|__| |__|__|

TREATMENT AND PLATELET COUNTS FROM THE BEGINNING OF "HEPARIN" THERAPY TO HIT SUSPICION

* **D0** = Date of HIT Suspicion

<table>
<tr><th>Day</th><th>Date</th><th>UFH</th><th>LMWH</th><th>Fondaparinux</th><th>Platelets (G/l)</th></tr>
<tr><td>D-0</td><td>|__|__| |__|__|
|__|__|</td><td>|__|</td><td>|__|</td><td>|__|</td><td>|__|__|__|</td></tr>
<tr><td>D-1</td><td>|__|__| |__|__|
|__|__|</td><td>|__|</td><td>|__|</td><td>|__|</td><td>|__|__|__|</td></tr>
<tr><td>D-2</td><td>|__|__| |__|__|
|__|__|</td><td>|__|</td><td>|__|</td><td>|__|</td><td>|__|__|__|</td></tr>
<tr><td>D-3</td><td>|__|__| |__|__|
|__|__|</td><td>|__|</td><td>|__|</td><td>|__|</td><td>|__|__|__|</td></tr>
<tr><td>D-4</td><td>|__|__| |__|__|
|__|__|</td><td>|__|</td><td>|__|</td><td>|__|</td><td>|__|__|__|</td></tr>
<tr><td>D-5</td><td>|__|__| |__|__|
|__|__|</td><td>|__|</td><td>|__|</td><td>|__|</td><td>|__|__|__|</td></tr>
<tr><td>D-6</td><td>|__|__| |__|__|
|__|__|</td><td>|__|</td><td>|__|</td><td>|__|</td><td>|__|__|__|</td></tr>
<tr><td>D-7</td><td>|__|__| |__|__|
|__|__|</td><td>|__|</td><td>|__|</td><td>|__|</td><td>|__|__|__|</td></tr>
<tr><td>D-8</td><td>|__|__| |__|__|
|__|__|</td><td>|__|</td><td>|__|</td><td>|__|</td><td>|__|__|__|</td></tr>
<tr><td>D-9</td><td>|__|__| |__|__|
|__|__|</td><td>|__|</td><td>|__|</td><td>|__|</td><td>|__|__|__|</td></tr>
<tr><td>D-10</td><td>|__|__| |__|__|
|__|__|</td><td>|__|</td><td>|__|</td><td>|__|</td><td>|__|__|__|</td></tr>
<tr><td>D-11</td><td>|__|__| |__|__|
|__|__|</td><td>|__|</td><td>|__|</td><td>|__|</td><td>|__|__|__|</td></tr>
<tr><td>D-12</td><td>|__|__| |__|__|
|__|__|</td><td>|__|</td><td>|__|</td><td>|__|</td><td>|__|__|__|</td></tr>
<tr><td>D-13</td><td>|__|__| |__|__|
|__|__|</td><td>|__|</td><td>|__|</td><td>|__|</td><td>|__|__|__|</td></tr>
<tr><td>D-14</td><td>|__|__| |__|__|
|__|__|</td><td>|__|</td><td>|__|</td><td>|__|</td><td>|__|__|__|</td></tr>
<tr><td>D-15</td><td>|__|__| |__|__|
|__|__|</td><td>|__|</td><td>|__|</td><td>|__|</td><td>|__|__|__|</td></tr>
<tr><td>D-16</td><td>|__|__| |__|__|
|__|__|</td><td>|__|</td><td>|__|</td><td>|__|</td><td>|__|__|__|</td></tr>
<tr><td>D-17</td><td>|__|__| |__|__|
|__|__|</td><td>|__|</td><td>|__|</td><td>|__|</td><td>|__|__|__|</td></tr>
</table>

**D-18**	|__|__| |__|__| |__|__|	|__|	|__|	|__|	|__|__|__|
**D-19**	|__|__| |__|__| |__|__|	|__|	|__|	|__|	|__|__|__|
**D-20**	|__|__| |__|__| |__|__|	|__|	|__|	|__|	|__|__|__|
**D-21**	|__|__| |__|__| |__|__|	|__|	|__|	|__|	|__|__|__|
**D-22**	|__|__| |__|__| |__|__|	|__|	|__|	|__|	|__|__|__|
**D-23**	|__|__| |__|__| |__|__|	|__|	|__|	|__|	|__|__|__|
**D-24**	|__|__| |__|__| |__|__|	|__|	|__|	|__|	|__|__|__|
**D-25**	|__|__| |__|__| |__|__|	|__|	|__|	|__|	|__|__|__|
**D-26**	|__|__| |__|__| |__|__|	|__|	|__|	|__|	|__|__|__|

**D-27**	|__|__| |__|__| |__|__|	|__|	|__|	|__|	|__|__|__|
**D-28**	|__|__| |__|__| |__|__|	|__|	|__|	|__|	|__|__|__|
**D-29**	|__|__| |__|__| |__|__|	|__|	|__|	|__|	|__|__|__|
**D-30**	|__|__| |__|__| |__|__|	|__|	|__|	|__|	|__|__|__|
**D-x**	|__|__| |__|__| |__|__|	|__|	|__|	|__|	|__|__|__|

HIT Biological tests	
First test performed	
______1. Date of the test:	|__|__| |__|__| |__|__|
2. Type of test and result:	
2.1. PAT..	☐1 Positive ☐ 2 Negative ☐3 Not done
2.2. ELISA	☐1 Positive ☐ 2 Negative ☐3 Not done
2.3. Serotonin release assay.....................................	☐1 Positive ☐ 2 Negative ☐3 Not done
2.4 Particle gel immunoassay...................................	☐1 Positive ☐ 2 Negative ☐3 Not done
Was a second test performed?	☐1 YES ☐2 NO
If yes, 1. Date of the test:	|__|__| |__|__| |__|__|
2. Type of test and result:	
2.1. PAT..	☐1 Positive ☐ 2 Negative ☐3 Not done
2.2. ELISA	☐1 Positive ☐ 2 Negative ☐3 Not done
2.3. Serotonin release assay.....................................	☐1 Positive ☐ 2 Negative ☐3 Not done
2.4 Particle gel immunoassay...................................	☐1 Positive ☐ 2 Negative ☐3 Not done
Was a third test performed?	☐1 YES ☐2 NO
If yes, 1. Date of the test:	|__|__| |__|__| |__|__|
2. Type of test and result:	
2.1. PAT..	☐1 Positive ☐ 2 Negative ☐3 Not done
2.2. ELISA	☐1 Positive ☐ 2 Negative ☐3 Not done
2.3. Serotonin release assay.....................................	☐1 Positive ☐ 2 Negative ☐3 Not done
2.4 Particle gel immunoassay...................................	☐1 Positive ☐ 2 Negative ☐3 Not done
Do you confirm at this time the diagnosis of HIT?	☐1 Yes ☐2 No ☐3 Possible

BIOLOGICAL DATA

FIRST HIT TEST

Date of test performance |__|__| |__|__| |__|__|

	Test	
❑	1. PAT	
	1.1. Number of normal blood donor samples:<br>1.2. Verification of platelet sensitivity to HIT antibodies :<br>1.3. Best percentage sensitivity observed	|__|__| |__|__| |__|__|__| %
❑	2. Serotonin release assay	
	2.1. Number of normal blood donor samples:<br>2.2. Verification of platelet sensitivity to HIT antibodies<br>2.3. Best percentage sensitivity observed	|__|__| |__|__| |__|__|__| %
❑	3. ELISA	
	3.1. Type of kit: ……………………………………<br>3.2. Result (OD) :<br>3.3. Cut-off	|__|,|__|__|__| |__|,|__|__|__|
❑	4. Particle gel immunoassay	☐1 Positive ☐2 Negative<br>☐3 Not done

BIOLOGICAL DATA

SECOND HIT TEST

Date of test performance |__|__| |__|__| |__|__|

	Tests	
❑	1. PAT	
	1.4. Number of normal blood donor samples:<br>1.5. Verification of platelet sensitivity to HIT antibodies :<br>1.6. Best percentage sensitivity observed	|__|__| |__|__| |__|__|__| %
❑	2. Serotonin release assay	

	2.1. Number of normal blood donor samples:<br>2.2. Verification of platelet sensitivity to HIT antibodies<br>2.3. Best percentage sensitivity observed	|__|__| |__|__| |__|__|__| %
❑	3. ELISA	
	3.1. Type of kit : ……………………………………<br>3.2. Result (OD) :<br>3.3. Cut-off	|__|,|__|__|__| |__|,|__|__|__|
❑	4. Particle gel immunoassay	☐1 Positive ☐ 2 Negative<br>☐3 Not done

BIOLOGICAL DATA

THIRD HIT TEST

Date Of Test performance |__|__| |__|__| |__|__|

	Tests	
❑	1. PAT	
	1.7. Number of normal blood donor samples:<br>1.8. Verification of platelet sensitivity to HIT antibodies :<br>1.9. Best percentage sensitivityobserved	|__|__| |__|__| |__|__|__| %
❑	2. Serotonin release assay	
	2.1. Number of normal blood donor samples:<br>2.2. Verification of platelet sensitivity to HIT antibodies<br>2.3. Best percentage sensitivity observed	|__|__| |__|__| |__|__|__| %
❑	3. ELISA	
	3.1. Type of kit: ……………………………………<br>3.2. Result (OD) :<br>3.3. Cut-off	|__|,|__|__|__| |__|,|__|__|__|
❑	4. Particle gel immunoassay	☐1 Positive ☐ 2 Negative<br>☐3 Not done

CLINICAL EVOLUTION FROM HIT SUSPICION TO HOSPITAL DISCHARGE

- Heparin continued ☐1Yes ☐2 No
  Normalization of platelet count ☐1Yes ☐2 No

- Definitive withdrawal of heparin ☐1Yes ☐2 No
  Normalization of platelet count ☐1Yes ☐2 No

- Heparin reintroduced after short withdrawal ☐1Yes ☐2 No
  Normalization of platelet count ☐1Yes ☐2 No

- Were the drugs inducing thrombocytopenia stopped? ☐1Yes ☐2 No

- Did thrombotic complications occur? ☐1Yes ☐2 No

- Did bleeding complications occur ? : ☐1Yes ☐2 No

  If yes :

  - requiring blood transfusion ☐1Yes ☐2 No
  - requiring surgery ☐1Yes ☐2 No

    Date of bleeding (DDMMYY) |__|__| |__|__| |__|__|

  - Did the bleeding lead to a fatal event ? ☐1Yes ☐2 No

TREATMENT AND PLATELET COUNTS SINCE HIT SUSPICION

- D0 = Date of HIT Suspicion

<table>
<tr><th>Day</th><th>Date</th><th>UFH</th><th>LMWH</th><th>Fondaparinux</th><th>Danaparoid</th><th>Lepirudin</th><th>Oral anticoagulant</th><th>Other</th><th>Platelet count</th></tr>
<tr><td>D 0</td><td>|__|__| |__|__| |__|__|</td><td>|__|</td><td>|__|</td><td>|__|</td><td>|__|</td><td>|__|</td><td>|__|</td><td>|__|</td><td>|__|__|__|</td></tr>
<tr><td>D +1</td><td>|__|__| |__|__| |__|__|</td><td>|__|</td><td>|__|</td><td>|__|</td><td>|__|</td><td>|__|</td><td>|__|</td><td>|__|</td><td>|__|__|__|</td></tr>
<tr><td>D+2</td><td>|__|__| |__|__| |__|__|</td><td>|__|</td><td>|__|</td><td>|__|</td><td>|__|</td><td>|__|</td><td>|__|</td><td>|__|</td><td>|__|__|__|</td></tr>
<tr><td>D+3</td><td>|__|__| |__|__| |__|__|</td><td>|__|</td><td>|__|</td><td>|__|</td><td>|__|</td><td>|__|</td><td>|__|</td><td>|__|</td><td>|__|__|__|</td></tr>
<tr><td>D+4</td><td>|__|__| |__|__| |__|__|</td><td>|__|</td><td>|__|</td><td>|__|</td><td>|__|</td><td>|__|</td><td>|__|</td><td>|__|</td><td>|__|__|__|</td></tr>
<tr><td>D+5</td><td>|__|__| |__|__| |__|__|</td><td>|__|</td><td>|__|</td><td>|__|</td><td>|__|</td><td>|__|</td><td>|__|</td><td>|__|</td><td>|__|__|__|</td></tr>
<tr><td>D+6</td><td>|__|__| |__|__| |__|__|</td><td>|__|</td><td>|__|</td><td>|__|</td><td>|__|</td><td>|__|</td><td>|__|</td><td>|__|</td><td>|__|__|__|</td></tr>
<tr><td>D+7</td><td>|__|__| |__|__| |__|__|</td><td>|__|</td><td>|__|</td><td>|__|</td><td>|__|</td><td>|__|</td><td>|__|</td><td>|__|</td><td>|__|__|__|</td></tr>
<tr><td>D+8</td><td>|__|__| |__|__| |__|__|</td><td>|__|</td><td>|__|</td><td>|__|</td><td>|__|</td><td>|__|</td><td>|__|</td><td>|__|</td><td>|__|__|__|</td></tr>
<tr><td>D+9</td><td>|__|__| |__|__| |__|__|</td><td>|__|</td><td>|__|</td><td>|__|</td><td>|__|</td><td>|__|</td><td>|__|</td><td>|__|</td><td>|__|__|__|</td></tr>
<tr><td>D+10</td><td>|__|__| |__|__| |__|__|</td><td>|__|</td><td>|__|</td><td>|__|</td><td>|__|</td><td>|__|</td><td>|__|</td><td>|__|</td><td>|__|__|__|</td></tr>
<tr><td>D+11</td><td>|__|__| |__|__| |__|__|</td><td>|__|</td><td>|__|</td><td>|__|</td><td>|__|</td><td>|__|</td><td>|__|</td><td>|__|</td><td>|__|__|__|</td></tr>
<tr><td>D+12</td><td>|__|__| |__|__| |__|__|</td><td>|__|</td><td>|__|</td><td>|__|</td><td>|__|</td><td>|__|</td><td>|__|</td><td>|__|</td><td>|__|__|__|</td></tr>
<tr><td>D+13</td><td>|__|__| |__|__| |__|__|</td><td>|__|</td><td>|__|</td><td>|__|</td><td>|__|</td><td>|__|</td><td>|__|</td><td>|__|</td><td>|__|__|__|</td></tr>
<tr><td>D+14</td><td>|__|__| |__|__| |__|__|</td><td>|__|</td><td>|__|</td><td>|__|</td><td>|__|</td><td>|__|</td><td>|__|</td><td>|__|</td><td>|__|__|__|</td></tr>
<tr><td>D+15</td><td>|__|__| |__|__| |__|__|</td><td>|__|</td><td>|__|</td><td>|__|</td><td>|__|</td><td>|__|</td><td>|__|</td><td>|__|</td><td>|__|__|__|</td></tr>
<tr><td>D+16</td><td>|__|__| |__|__| |__|__|</td><td>|__|</td><td>|__|</td><td>|__|</td><td>|__|</td><td>|__|</td><td>|__|</td><td>|__|</td><td>|__|__|__|</td></tr>
<tr><td>D+17</td><td>|__|__| |__|__| |__|__|</td><td>|__|</td><td>|__|</td><td>|__|</td><td>|__|</td><td>|__|</td><td>|__|</td><td>|__|</td><td>|__|__|__|</td></tr>
<tr><td>D+18</td><td>|__|__| |__|__| |__|__|</td><td>|__|</td><td>|__|</td><td>|__|</td><td>|__|</td><td>|__|</td><td>|__|</td><td>|__|</td><td>|__|__|__|</td></tr>
<tr><td>D+19</td><td>|__|__| |__|__| |__|__|</td><td>|__|</td><td>|__|</td><td>|__|</td><td>|__|</td><td>|__|</td><td>|__|</td><td>|__|</td><td>|__|__|__|</td></tr>
</table>

**D+20**	|__|__| |__|__| |__|__|	|__|	|__|	|__|	|__|	|__|	|__|	|__|	|__|__|__|
**D+21**	|__|__| |__|__| |__|__|	|__|	|__|	|__|	|__|	|__|	|__|	|__|	|__|__|__|
**D+22**	|__|__| |__|__| |__|__|	|__|	|__|	|__|	|__|	|__|	|__|	|__|	|__|__|__|
**D+23**	|__|__| |__|__| |__|__|	|__|	|__|	|__|	|__|	|__|	|__|	|__|	|__|__|__|
**D+24**	|__|__| |__|__| |__|__|	|__|	|__|	|__|	|__|	|__|	|__|	|__|	|__|__|__|
**D+25**	|__|__| |__|__| |__|__|	|__|	|__|	|__|	|__|	|__|	|__|	|__|	|__|__|__|
**D+26**	|__|__| |__|__| |__|__|	|__|	|__|	|__|	|__|	|__|	|__|	|__|	|__|__|__|
**D+27**	|__|__| |__|__| |__|__|	|__|	|__|	|__|	|__|	|__|	|__|	|__|	|__|__|__|
**D+28**	|__|__| |__|__| |__|__|	|__|	|__|	|__|	|__|	|__|	|__|	|__|	|__|__|__|
**D+29**	|__|__| |__|__| |__|__|	|__|	|__|	|__|	|__|	|__|	|__|	|__|	|__|__|__|
**D+30**	|__|__| |__|__| |__|__|	|__|	|__|	|__|	|__|	|__|	|__|	|__|	|__|__|__|

**D+X**	|__|__| |__|__| |__|__|	|__|	|__|	|__|	|__|	|__|	|__|	|__|	|__|__|__|

End of the study

1. Date of the end of follow-up: |__|__| |__|__| |__|__|

2. Death : ☐1 YES ☐2 NO

IF yes 1 date : |__|__| |__|__| |__|__|
Cause : ……………………………… ………………………………….
…………………………………………………………………….

3. Did you find any cause other than HIT to explain the thrombocytopenia episode?
☐1YES ☐2 NO

If yes, which cause? ……………………………….
……………………………….
……………………………….

4. At the end of hopitalization, was the patient discharged with a final diagnosis of HIT?
YES ☐1 ☐2 NO ☐2 POSSIBLE

Additional comments: YES ☐1 ☐2 NO
If yes : ……………………………………………………
……………………………………………………
……………………………………………………

Experts

1) Do you confirm the diagnosis of HIT ?

	HIT confirmed ☐ Yes ☐ No ☐ Possible

2) Result of centralized performance of serotonin release assay:

Positive ☐ Negative ☐ Doubtful ☐ Non-specific Platelet Activation ☐

Number of normal blood donor samples	☐ [--]
Verification of platelet sensitivity to HIT antibodies	☐ [--]
Best percentage sensitivity of serotonin release assay obtained	
Low concentration of heparin	☐ [---] %
Dose of heparin (IU)	☐ [-.-] UI
- first high concentration of heparin	☐ [---] %
Dose of heparin (IU)	☐ [---] UI
- second high concentration of heparin	☐ [---] %
Dose of heparin (UI)	☐ [---] UI

3) What was it your final opinion?

- HIT diagnosis confirmed ☐

- HIT diagnosis possible ☐
- HIT diagnosis excluded ☐
- Request for another serotonin release assay ☐
- Request for additional clinical data ☐
